# Prevalence of vomiting and nausea and associated factors after chronic and acute gluten exposure in celiac disease

**DOI:** 10.1186/s12876-023-02934-w

**Published:** 2023-09-06

**Authors:** Iida Ahonen, Pilvi Laurikka, Sara Koskimaa, Heini Huhtala, Katri Lindfors, Katri Kaukinen, Kalle Kurppa, Laura Kivelä

**Affiliations:** 1 Celiac Disease Research Center, Faculty of Medicine and Health Technology, Tampere University, Tampere, Finland; 2 Department of Internal Medicine, Tampere University Hospital, Tampere, Finland; 3 Tampere Center for Child, Adolescent and Maternal Health Research, Tampere University and Tampere University Hospital, Tampere, Finland; 4 Faculty of Social Sciences, Tampere University, Tampere, Finland; 5 The University Consortium of Seinäjoki, Seinäjoki, Finland; 6 Children’s Hospital and Pediatric Research Center, University of Helsinki and Helsinki University Hospital, Helsinki, Finland

**Keywords:** Celiac disease, Vomiting, Nausea, Gluten challenge

## Abstract

**Background:**

Vomiting and nausea seem to be relatively specific symptoms related to gluten ingestion in treated celiac disease. However, the overall prevalence and associated factors of these symptoms after chronic gluten exposure at celiac disease diagnosis and acute re-exposure during gluten challenge remain obscure.

**Methods:**

Medical data on 815 adult celiac disease patients were collected at diagnosis from the medical records and through supplementary interviews. An additional 74 patients underwent a three-day (10 g/day) gluten challenge (wheat, barley, rye or a combination of the three grains) while in remission. Prevalence of vomiting/nausea and associated factors were evaluated in both cohorts. A literature review was conducted to summarize earlier studies.

**Results:**

Twenty-eight (3%) patients presented with vomiting at diagnosis. They were less often screen-detected and suffered from extra-intestinal symptoms, and had more often abdominal pain (71% vs. 49%, *p* = 0.021), diarrhea (61% vs. 40%, *p* = 0.031), weight loss (36% vs. 17%, *p* = 0.019) and childhood symptoms (61% vs. 33%, *p* = 0.002) than those without vomiting (*n* = 787). The groups were comparable in other clinical-demographic data and in genetic, serological, and histological findings. Short-term gluten challenge provoked vomiting/nausea in 14/74 (19%) patients. They consumed gluten-free oats less often than those without these symptoms (64% vs. 92%, *p* = 0.017), whereas the groups did not differ in clinical-demographic features at diagnosis, presence of comorbidities, duration of gluten-free diet, or in other symptoms or grain used ingested during the challenge. According to the literature, prevalence of vomiting/nausea at celiac disease diagnosis has varied 3–46% and during gluten challenge 13–61%.

**Conclusions:**

In chronic gluten exposure at celiac disease diagnosis, vomiting was associated with other gastrointestinal symptoms and onset of symptoms already in childhood, whereas regular consumption of oats may increase the tolerance against vomiting/nausea after acute re-exposure in treated celiac disease.

## Background

Celiac disease (CeD) is an immune-mediated condition in which dietary gluten causes small-bowel mucosal damage and a variety of gastrointestinal and extraintestinal complaints [1]. These symptoms may arise from several organ systems and their severity ranges from asymptomatic to life-threatening. Nausea and vomiting are classic gastrointestinal symptoms of CeD and are reported particularly in the youngest pediatric patients [2, 3], whereas the prevalence and associated factors of these symptoms in adults remain poorly defined. Inadequate recognition of possibly CeD-related symptoms results in significant underdiagnosis and long diagnostic delay [4, 5] and subsequently predispose patients to severe complications such as impaired bone health, infertility, and malignancy [6], as well as impaired quality of life and increased use of health care services [5].

Symptoms during a “normal” diet before CeD diagnosis can be considered to be caused by chronic gluten exposure. Similarly, as chronic ingestion during untreated CeD, acute re-exposure to gluten in patients placed already on a gluten-free diet (GFD) can cause diverse symptoms of varying severity [7–9]. Intentional gluten challenge has an important role in CeD diagnostics when GFD has been started prior to the diagnostic testing, but also in the development of novel diagnostic methods and medications for CeD [10]. However, it may be challenging to distinguish whether the symptoms during the challenge are actually caused by gluten or by some other dietary factors and thus be unrelated to CeD [11]. Interestingly, recent evidence from treated CeD patients re-exposed to gluten suggests that vomiting and nausea are relatively CeD-specific symptoms [9, 12, 13]. Knowing more about the factors associated with these symptoms could increase the understanding of the mechanisms of different reactions to gluten in CeD and help to develop more accurate patient-related outcome measures, especially for CeD drug studies.

Our aims were to investigate the prevalence and associated factors of vomiting and nausea 1) after chronic gluten exposure at the time of CeD diagnosis and 2) after acute re-exposure during short-term gluten challenge in treated CeD. We moreover conducted a literature review to summarize earlier studies published on these topics.

## Methods

### Patients and study design

The study was conducted at Tampere University and Tampere University Hospital. It comprised two separate groups of biopsy-proven adult (age ≥ 18 years) CeD patients:

1. To study chronic gluten exposure in untreated CeD, previously diagnosed CeD patients were recruited with the help of national and local patient organizations and through newspaper advertisements [14]. All participants (*n* = 993) were interviewed by a study nurse or physician with expertise in CeD and patient records were obtained with due permission. After excluding patients with unconfirmed CeD diagnosis or missing clinical data at diagnosis (*n* = 178), the final cohort comprised 815 patients.
2. To study acute re-exposure to gluten, asymptomatic CeD patients who had been following a strict GFD ≥ 1 year were recruited via advertisements in Celiac Society newsletters [15]. Strict GFD was defined as no dietary lapses and negative CeD serology at the first study visit. Patients with unconfirmed diagnosis, immunosuppressant medication, severe complication or co-morbidity such as refractory CeD, small-bowel malignancy, and diabetes mellitus with poor glycemic control, were excluded. Altogether 74 patients comprised the final sub-cohort and underwent a three-day gluten challenge. They consumed 10 g gluten per day in the form of bread or porridge containing either wheat, barley, rye or a combination of all three grains. This approach was chosen as the grain type could affect the immunological and clinical response provoked by the challenge [16], and since all of these grains are commonly used as part of everyday gluten-containing Finnish diet. The grain type was randomly allocated to the participants. All patients attended a post-challenge visit on day six.

### Study variables and outcomes

#### Chronic gluten exposure

Data were collected retrospectively on demographics, diagnostic approach, symptoms and their duration, severity of duodenal villous atrophy, and celiac antibody levels at diagnosis, comorbidities such as type 1 diabetes mellitus, thyroidal disease or gastrointestinal disease, and presence of CeD in first-degree relatives. CeD-associated genetics were determined during the study visit.

Clinical presentation was classified as gastrointestinal symptoms (e.g. abdominal pain, diarrhea and bloating), extra-intestinal complaints (e.g. joint pain, dermatological symptoms, and anemia), or screening among at-risk groups such as type 1 diabetes or CeD in the family. Duration of symptoms before diagnosis was classified as < 1, 1–5, 6–10 or > 10 years, and possible symptoms already in childhood were assessed separately.

The severity of small-bowel mucosal atrophy at diagnosis was collected from pathology reports and further classified into partial, subtotal or total, these corresponding approximately to the Marsh-Oberhuber classification IIIa-c [17]. IgA-class endomysial antibodies (EmA) were determined in clinical practice by indirect immunofluorescence [18]. A dilution of ≤ 1:5 was considered positive, and positive samples were further diluted up to 1:4000 or until negative. In the case of selective IgA deficiency, the corresponding IgG-class antibodies were assessed. Transglutaminase 2 antibody values (TGA) are not reported here since they have been measured with several commercial assays with varying reference values in clinical practice and the data about the used test kit was not available.

Blood samples were drawn from each participant and CeD-associated human leukocyte antigen (HLA) genotype was determined either by a commercial HLA typing kit (Olerup SSP low-resolution kit, Olerup SSP AB, Saltsjöbaden, Sweden, or DELFIA Celiac Disease Hybridization Assay Kit, PerkinElmer Life and Analytical Sciences, Wallac Oy, Turku, Finland) or using chemistry-based HLA typing with tagging single nucleotide polymorphisms (SNPs). HLA DQ2.5/DQ2.5 and HLA DQ2.5/DQ2.2 were considered high genetic risk for CeD [19].

#### Acute gluten re-exposure

Presence of CeD in first-degree relatives and comorbidities, duration of GFD before the study visit, and regular (≥ once a month) use of gluten-free oats in the diet were assessed during the study visit. Height and weight were measured to calculate body-mass index (kg/m^2^), and EmA and TGA were evaluated from blood samples*.* EmA were determined as described in detail above, and IgA-class TGA were measured by enzyme-linked immunosorbent assay (Celikey®, Phadia, GmbH, Freiburg, Germany). In case of selective IgA deficiency, IgG-class antibodies were assessed. Data on CeD diagnosis were verified from medical records and clinical presentation was categorized as gastrointestinal symptoms, extra-intestinal complaints, and risk-group screen-detected similarly as in chronic gluten exposure.

On a post-challenge visit on day six, the patients were interviewed about possible symptoms during the challenge and residual symptoms persisting after the challenge, and EmA and TGA were measured again.

#### Systematic literature review

PubMed database was searched for articles from January 2000 to September 2022 reporting vomiting and/or nausea in adults with CeD. Only original studies written in English with an available abstract were included. The search was conducted using the terms (“celiac disease” or “coeliac disease”) and (“symptoms” or “features” or “characteristics” or “presentation”) and (“gastrointestinal” or “gastro-intestinal” or “vomiting” or “nausea”) and (“gluten challenge” or “gluten exposure”) in the title or abstract. Additional known relevant studies were added.

### Statistical analyses

Categorical variables are reported as numbers and percentages and compared using χ^2^ or Fisher’s exact test. As most numerical values were non-normally distributed, they are reported as medians with quartiles and compared with Kruskal–Wallis or Mann–Whitney U test. *P*-value < 0.05 was considered significant. All analyses were conducted with Statistics Package for the Social Sciences version 25 (IBM, Corporation, Armonk, NY).

### Ethical aspects

The ethical guidelines of the Declaration of Helsinki were followed. The Ethics Committee of the Pirkanmaa Hospital District approved the data collection. All study patients gave written informed consent and were aware of their opportunity to withdraw their consent to participate at any time without explanation. Gluten challenge products were given to participants free of charge.

## Results

### Chronic gluten exposure

**Fig. 1:**
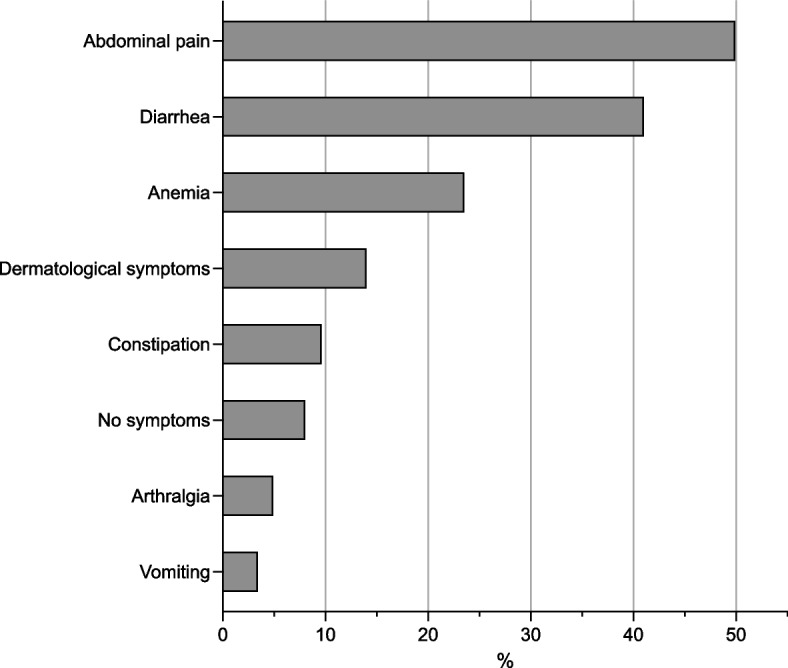
Prevalence of vomiting and other symptoms in untreated celiac disease at the time of diagnosis (*n* = 815). Data about nausea was not available. Asymptomatic patients were found by screening at-risk groups

Altogether 28 (3%) out of 815 patients reported vomiting and it was the least common of the CeD related symptoms elicited at CeD diagnosis (Fig. 1). Data about nausea at diagnosis was not available.

Patients who experienced vomiting were found less often by risk-group screening and due to extraintestinal complaints, and had more often abdominal pain, diarrhea, and weight loss than those without vomiting (Table 1). Additionally, those with vomiting had already suffered from symptoms more often in childhood and the duration of symptoms before the CeD diagnosis was more often either < 1 year or > 10 years. The groups did not differ in age or sex distribution, time of diagnosis, severity of villous atrophy, celiac antibody levels, HLA risk, family history or comorbidities at diagnosis (Table 1).

**Table 1:** Comparison of clinical, serological, and histological parameters and HLA distribution between celiac disease patients with and without vomiting after chronic gluten exposure at celiac disease diagnosis (*n* = 815)

	**Vomiting at diagnosis**		
	**Yes, *n* = 28**	**No, *n* = 787**	***P* value**
Females, %	89	77	0.127
Age at diagnosis, median (IQR), years	44 (34, 53)	38 (30, 52)	0.182
Year of diagnosis, median (IQR)	2000 (1994, 2004)	1995 (1988, 2004)	0.085
Other symptoms at diagnosis, %			
Abdominal pain	71	49	**0.021**
Diarrhea	61	40	**0.031**
Weight loss	36	17	**0.019**
Arthralgia	7	5	0.643
Anemia	22	24	0.874
Dermatological symptoms	4	14	0.161
Clinical features at diagnosis, %			**0.002**
Gastrointestinal	96	65	
Extraintestinal	0	21	
Screen-detected	4	14	
Duration of symptoms, years, %			**0.018**
< 1	36	20	
1–5	18	35	
6–10	0	11	
> 10	46	34	
Any symptoms in childhood, %	61	33	**0.002**
Severity of villous atrophy at diagnosis, %			0.670
Partial	37	35	
Subtotal	44	39	
Total	19	26	
EmA at diagnosis, median (IQR), titer^a^	1:50 (1:10, 1:100)	1:50 (1:200, 1:500)	0.192
High-risk HLA^b^, %	30	22	0.369
Family history for celiac disease^c^, %	75	65	0.256
Comorbidities, %			
Type 1 diabetes	0	2	1.000
Thyroid disease	18	14	0.585
Other gastrointestinal disease^d^	21	18	0.614

Data were available on > 75% patients in each variable, except in ^a^*n* = 175; ^b^DQ2.5/2.5 or DQ2.5/2.2; ^c^First-degree relatives; ^d^E.g. inflammatory bowel disease, irritable bowel syndrome, gastroesophageal reflux

Values in bold face denote statistical significance

*EmA* Endomysial antibodies, *HLA* Human leukocyte antigen, *IQR* Interquartile range

### Acute gluten re-exposure

Vomiting was reported by 5 (7%) and nausea by 9 (12%) out of the 74 CeD patients during the three-day gluten challenge. Presence of these symptoms was associated with less common use of gluten-free oats, whereas the groups did not differ in age or sex distribution, body mass index, clinical features at diagnosis or duration of the diet, family history of CeD, presence of comorbidities, type of grain used in the challenge, presence of other symptoms during or after the challenge or serology after the challenge (Table 2). Less common use of oats was not associated with symptoms other than vomiting/nausea provoked during the gluten challenge (data not shown).

**Table 2:** Comparison of clinical and celiac disease-related parameters among 74 treated patients experiencing / not experiencing vomiting and/or nausea during three-day acute gluten exposure

	**Vomiting/nausea during gluten challenge**		
	**Yes, *n* = 14**	**No, *n* = 60**	***P* value**
Current age, median (IQR), years	53 (38, 60)	54 (40, 64)	0.619
Duration of GFD, median (IQR), years	7 (3, 11)	8 (2, 17)	0.864
BMI, median (IQR), kg/m^2^	26 (23, 28)	25 (23, 28)	0.934
Females, %	86	72	0.497
Clinical features at diagnosis, %			0.784
Gastrointestinal	71	65	
Extra-intestinal	21	30	
Screen-detected	7	5	
Family history for celiac disease^a^, %	29	32	1.000
Any chronic comorbidity, %	57	57	1.000
Other autoimmune disease^b^, %	7	8	1.000
Regular use of oats^c^, %	64	92	**0.017**
Grain used in the challenge, %			0.112
Wheat	43	37	
Barley	0	25	
Rye	43	23	
Combination of wheat, barley and rye	14	16	
Other symptoms during gluten challenge			
Diarrhea/loose stools, %	21	20	1.000
Abdominal pain, %	21	27	1.000
Extra-intestinal symptoms, %	14	8	0.611
Residual symptoms^d^, %	21	25	1.000
EmA after the challenge, median (IQR), titer	0	0	1.000
TGA after the challenge, median (IQR), kU/L	0 (0, 2)	1 (0, 1)	0.944

Data were available on > 80% patients in each variable. Values in bold face denote statistical significance

*BMI* Body-mass index, *EmA* Endomysial antibodies, *GFD* Gluten-free diet, *IQR* Interquartile range, *TGA* Transglutaminase 2 antibodies

^a^First-degree relatives

^b^Type 1 diabetes or autoimmune thyroiditis

^c^ ≥ once a month

^d^Symptoms persisting three days after the challenge

### Literature review

The search identified 674 publications about chronic gluten exposure in CeD and 164 publications about acute gluten re-exposure. After excluding irrelevant publications based on title and abstract evaluation (*n* = 626), unclear research design (*n* = 4), and lack of full text (*n* = 2), 42 publications on chronic gluten exposure were assessed in detail and of these ten fulfilled the study criteria. Additional relevant studies were included, and the final analysis comprised 14 publications. Correspondingly, after excluding irrelevant publications based on title and abstract evaluation (*n* = 137), 27 full texts on acute re-exposure were assessed in detail and of these nine were included.

Studies evaluating symptoms after chronic gluten exposure at CeD diagnosis reported vomiting in 3–33%, nausea in 14–36%, and either vomiting or nausea in 12–46% of patients (Table 3). Sample size in the studies included varied between 31 and 1166, mean/median age of patients 30–70 years, and proportion of women 47–81%. Sixty-seven percent (*n* = 10) of the previous studies were retrospective and 33% (*n* = 5) prospective (Table 3).

**Table 3:** Prevalence of vomiting and nausea after chronic gluten exposure at celiac disease diagnosis in adult patients as found in the literature

Country, publication year	Patients^a^	Females	Age at dg, years	Vomiting	Nausea	Vomiting or nausea,
	n	%	Mean ± SD or median (Q_1_, Q_3_ or range)	%	%	%
*Volunteered celiac disease patients, retrospective*						
** Current study**	**815**	**77**	**44 (34, 53)**	**3**	**ND**	**ND**
*Celiac disease society members, retrospective*						
USA, 2003 [20]	134	81	45 ± 11^b^	ND	ND	46
*Patients found in clinical practice, retrospective*						
Italy, 2020 [21]	278	69	35 ± 12^b^	3	ND	ND
Brazil, 2019 [22]	240	67	38 ± 13^b^	10	36	ND
USA, 2018 [23]	250^c^	78	39 (18–76)	9	15	ND
Netherlands, 2016 [24]	412	66	40 (21, 58)^b^	5	16	ND
Iran, 2014 [25]	103	61	32 ± 11^b^	18	ND	ND
Pakistan, 2013 [26]	77	47	30 ± 13^b^	33	ND	ND
UK, 2007 [27]	105	67	54 (25–88)	5	ND	ND
UK, 2006 [28]	32	60	53 (23–86)	ND	ND	28
Italy, 2001 [29]	286	71	38 ± 5^b^	ND	ND	12
*Patients found in clinical practice, prospective*						
Italy, 2022 [30]	317	73	36 (18–76)^d^	ND	ND	21
Canada, 2016 [7]	105^e^	69	37 (27, 54)	9	ND	ND
Italy, 2012 [31]	1166	74	35 ± 11	4	ND	ND
Italy, 2012 [31]	59	58	70 ± 4	5	ND	ND
USA, 2011 [32]	31^f^	65	55 ± 15	4	14	ND

*ND* No data, *SD* Standard deviation

^a^Data available of symptoms

^b^Not specified whether childhood diagnoses were also included

^c^Only patients with gastrointestinal symptoms were included in the calculations

^d^Age of females

^e^Participation rate of invited patients (*n* = 182) was 58%

^f^Diagnosis based on seropositivity

Gluten challenge studies reported vomiting after acute gluten re-exposure in 8–44% and nausea in 13–61% of the patients (Table 4). Sample size varied between 15 and 295, mean/median age of study patients 28–50 years, proportion of women 60–88%, and mean/median duration of GFD before the re-exposure to gluten 4–12 years. Duration of gluten challenge/exposure ranged from a single dose up to six weeks and the daily amount of gluten consumed was 2–14 g in varying forms (Table 4).

**Table 4:** Prevalence of vomiting and nausea after acute gluten exposure in treated celiac disease patients

Country, year	Patients	Females	Current age, yr	Duration of GFD, yr	Vomiting	Nausea
	n	%	Mean ± SD or Median (Q_1_, Q_3_ or range)	Mean ± SD or Median (Q_1_, Q_3_ or range)	%	%
*Gluten challenge: 10–14 g per day in bread or porridge for 3 days*						
**Current study**	**74**	**75**	**54 (39,63)**	**7 (3,14)**	**7**	**12**
Spain, 2018 [33]	15	60	28 ± 4	4 ± 2	ND	13
*Gluten challenge: 6 g of single gluten bolus*						
Multicenter^a^, 2020 [9]	295	69	43 ± 15	6 (3,10)^b^	21	61
Australia, 2020 [12]	36	69	42 (34,54)	5 (2,8)^b^	44	61
Australia, 2019 [13]	25	88	39 ± 16	4 (1–20)	24	40
*Gluten challenge: 3 g gluten in single suspension*						
USA, 2022 [34]	20	75	49 (37, 56)	7 (6, 11)	ND	30
*Gluten challenge: 2–3 g per day in biscuit or bread for 6 weeks*						
USA, 2022 [35]^c^	22	67	45	ND	8	21
Finland, 2021 [36]	38	74	43 ± 14	ND	21	18
USA, 2013 [37]	43	66	50 ± 10	6 ± 5	ND	19
Finland, 2014 [38]	21	81	50 (19–71)	12 (1–39)	14	19

*GFD* Gluten-free diet, *ND* No data

^a^USA, Australia and New Zealand

^b^Years from celiac disease diagnosis

^c^No data about the exact form of gluten

## Discussion

### Chronic gluten exposure

Vomiting was present in 3% of untreated CeD patients and was the least frequently reported complaint in the present study. For comparison, figures as high as 33% have been reported previously (Table 3). As a plausible explanation, varying screening practices and awareness of CeD between countries could affect the diagnostic delay and therefore clinical presentation [39]. However, part of the variation could also be due to methodological issues. For example, the retrospective design and self-reporting the symptoms in the present and many earlier studies increase the risk of recall bias as well as unsystematic symptom definition and reporting. Additionally, varying prevalence of other conditions often presenting with vomiting, such as intestinal parasitemia, could lead to deceptive geographic differences [40, 41].

Logically, the presence of vomiting at CeD diagnosis was associated with other gastrointestinal symptoms—abdominal pain, diarrhea, and weight loss, whereas it was less common among screen-detected patients, who are more often asymptomatic. Additionally, vomiting was overrepresented in subjects with particularly short or long diagnostic delay and in those who had already had symptoms in childhood. This may be due to the fact that, on the one hand, vomiting is often considered an alarm symptom necessitating prompt diagnostic evaluations but, on the other hand, it may also go unrecognized as a sign of CeD. Somewhat in contrast with our earlier findings [42, 43], neither histologic nor serologic findings were associated with vomiting. Altogether, the relationship between clinical and histological presentation of CeD is a complex and highly individual variable [44, 45] More research on this issue is needed. Some studies have also reported increased frequency of vomiting among elderly patients [29] and females with CeD [22], but no such associations were seen here.

### Acute gluten re-exposure

Vomiting was provoked in 7% and nausea in 12% of patients re-exposed to gluten while following a GFD. Prevalence of these symptoms in acute exposure has previously varied significantly (Table 4), possible explanations for this being again partly methodological and patient related. Also, the frequency and amount of gluten consumed in the challenge may have an effect. In general, the highest prevalence of vomiting and nausea was with a gluten bolus of six grams. Tye-Din et al. have previously reported vomiting to become more common after repeated gluten challenge [9], whereas this was not seen after daily administration in our literature review possibly reflecting the attenuation of the response (Table 4). However, the frequency but also the instant of recording could affect the prevalence, as the onset of symptoms after gluten exposure in CeD has ranged from 10 min to 48 h [7]. Additionally, the form in which gluten is ingested may have an effect. Although e.g., bread and porridge resemble more real-life exposure than pure gluten bolus, they may contain also other symptom-causing agents such as FODMAPs [11].

CeD patients reporting vomiting or nausea during the challenge consumed less often oats in their GFD than did those without these symptoms. Since these complaints can be particularly disturbing for patients [9], it is possible that this patient group avoids oats for fear of symptoms. On the other hand, our findings could indicate that regular use of oats increases clinical tolerance, but the development of other symptoms than vomiting/nausea was not associated with the use of oats here. Possible beneficial effects of oats on gastrointestinal health in general could be explained by soluble fibers of oats, which promotes gut balance e.g. by increasing the growth of beneficial bacteria in microbiota and improving stool composition and frequency [46, 47]. We previously observed low intake of fiber to be associated with symptom persistence on GFD [48], and soluble fiber can alleviate symptoms in irritable bowel syndrome [49, 50]. Altogether, dietary restrictions in addition to GFD as well as possible gluten exposures and symptoms experienced should be routinely assessed during CeD follow-up as these could be associated with poor eating behavior, anxiety, and social restrictions [8, 51].

Other factors than consumption of oats did not predict vomiting/nausea during the challenge. In line with this, Tye-Din et al. reported no association between patients’ characteristics and acute clinical response; however, they found a change in serum interleukin-2 (IL-2) levels to be associated with age and genetics [9]. Acute gluten exposure has been associated with increased IL-2 particularly in patients with nausea and vomiting, supporting the gluten-specificity of these symptoms [9, 52, 53]. However, in contrast, Cartee et al. reported no difference in the prevalence of nausea after gluten and sham challenges [34]. In general, highlighting the role of individual factors, Stamnaes et al. found that low-grade mucosal inflammation in treated CeD patients was associated with more marked histological response to gluten challenge [54]. Further studies are again needed to elucidate the pathological mechanisms behind the diverse responses to gluten exposure in CeD patients [55, 56].

### Strengths and limitations

The main strengths of the sub-study considering untreated CeD and chronic gluten exposure are the large cohort of patients and the collection of comprehensive medical record data, which was supplemented in the interviews. The main limitations were the retrospective design and recruitment of the patients via CeD associations, which may have caused recall bias and selection. The strengths of the acute challenge sub-study are the prospective design, the moderate gluten dose and its form in basic groceries, which could be considered to mimic real-life exposures to gluten relatively well although the amount of gluten is likely higher. As a limitation, however, the effects of grain components other than gluten on the symptoms cannot be excluded. Furthermore, the challenge was not placebo-controlled and actual intake of gluten was self-reported.

## Conclusions

In the present study, vomiting was reported by 3% of untreated patients after chronic gluten exposure at CeD diagnosis and by 14% of the treated patients during acute gluten re-exposure. Vomiting at CeD diagnosis was associated with other gastrointestinal symptoms, either long or short diagnostic delay, and onset of symptoms already in childhood, whereas vomiting/nausea after acute gluten re-exposure was less common in those reporting regular consumption of oats. In addition to individual factors, the amount, duration, and frequency of gluten consumed could affect the development of symptoms. This emphasizes the need for standardized protocols for gluten challenge both in research use and in clinical practice. On the other hand, the tendency to experience symptoms in general could be associated with eating behavior, reminding the importance of assessing possible symptoms, gluten exposure, and overall diet during CeD follow-up.

## Data Availability

The datasets used and/or analysed during the current study available from the corresponding author on reasonable request.

## Abbreviations

CeD: Celiac disease
EmA: Endomysial antibodies
GFD: Gluten-free diet
HLA: Human leukocyte antigen
TGA: Transglutaminase 2 antibodies
